# ATP Consumption Is Coupled with Endocytosis in Exudated Neutrophils

**DOI:** 10.3390/ijms24109039

**Published:** 2023-05-20

**Authors:** Duo Wang, Zirui Zeng, Mengyue Shen, Ryuji Okazaki, Hironori Miyata, Tomo Yonezawa, Yasuhiro Yoshida

**Affiliations:** 1 Department of Radiobiology and Hygiene Management, Institute of Industrial Ecological Sciences, University of Occupational and Environmental Health, Japan, 1-1 Iseigaoka, Yahatanishi-ku, Kitakyushu 807-8555, Japan; 2 The First Department of Internal Medicine, School of Medicine, University of Occupational and Environmental Health, Japan, 1-1 Iseigaoka, Yahatanishi-ku, Kitakyushu 807-8555, Japan; 3 Department of Immunology and Parasitology, School of Medicine, University of Occupational and Environmental Health, Japan, 1-1 Iseigaoka, Yahatanishi-ku, Kitakyushu 807-8555, Japan; 4 Department of Medical Teaching, West China Center of Medical Sciences of Sichuan University, Chengdu 610041, China; 5 Laboratory Animal Research Center, University of Occupational and Environmental Health, Japan, 1-1 Iseigaoka, Yahatanishi-ku, Kitakyushu 807-8555, Japan; 6 Division of Functional Genomics and Therapeutic Innovation, Research Center for Advanced Genomics, Graduate School of Biomedical Sciences, Nagasaki University, 1-12-14 Sakamoto, Nagasaki 852-8523, Japan

**Keywords:** neutrophil, endocytosis, adenosine triphosphate (ATP), particulate matter, inflammatory cytokine, inflammasome

## Abstract

Neutrophil energy metabolism during phagocytosis has been previously reported, and adenosine triphosphate (ATP) plays a crucial role in endocytosis. Neutrophils are prepared by intraperitoneal injection of thioglycolate for 4 h. We previously reported a system established for measuring particulate matter endocytosis by neutrophils using flow cytometry. In this study, we utilized this system to investigate the relationship between endocytosis and energy consumption in neutrophils. A dynamin inhibitor suppressed ATP consumption triggered by neutrophil endocytosis. In the presence of exogenous ATP, neutrophils behave differently during endocytosis depending on ATP concentration. The inhibition of ATP synthase and nicotinamide adenine dinucleotide phosphate oxidase but not phosphatidylinositol-3 kinase suppresses neutrophil endocytosis. The nuclear factor kappa B was activated during endocytosis and inhibited by I kappa B kinase (IKK) inhibitors. Notably, IKK inhibitors restored endocytosis-triggered ATP consumption. Furthermore, data from the NLR family pyrin domain containing three knockout mice suggest that inflammasome activation is not involved in neutrophil endocytosis or concomitant ATP consumption. To summarize, these molecular events occur via endocytosis, which is closely related to ATP-centered energy metabolism.

## 1. Introduction

Air pollution caused by particulate matter (PM) of ≤2.5 microns in width (PM2.5) is a serious concern in many Asian countries. In China, it is estimated that PM2.5 contributes to roughly 32% of deaths in metropolitan areas, with cardiovascular disease, respiratory disease, and lung cancer being the major causes [1]. There are reports in Japan that when PM2.5 levels rise, the number of emergency transports related to respiratory diseases increases. One cohort study reported that a 10 μg/m^3^ increase in PM2.5 increased deaths from respiratory diseases by 16% and deaths from lung cancer by 24% [2], supporting the relationship between PM2.5 concentrations and respiratory diseases.

We previously reported the biological effects of air pollutants such as Asian sand dust (Kosa) and PM2.5 on preclinical animal models [3]. Asian sand dust, which consists of particles less than ten microns in width (PM10), induces systemic inflammation [4,5,6] and upregulates ovalbumin-induced neutrophils [7]. Toll-like receptors 2 and 4 are involved in PM-induced inflammation [8,9]. PM2.5, which causes neutrophil infiltration in the lungs, is a risk factor for inflammation and allergic diseases [10] and induces neutrophil inflammation and oxidative stress responses [8,11].

Neutrophils are one of the most abundant white blood cell types in the human peripheral blood. The key roles of neutrophils involve quickly responding to extracellular pathogens and helping to initiate the adaptive immune response [12]. During endocytosis, neutrophils form neutrophil extracellular traps to capture pathogens and release reactive oxygen species (ROS) to remove pathogens. We demonstrated that dynamin is required for neutrophil endocytosis of particulate matter [8], and following endocytosis, neutrophils produce inflammatory cytokines. At the beginning of endocytosis, endosomes are closed, and type I phosphoinositide 3-kinases (PI3Ks) play an important role in this step [13]. The next step is phagosome maturation, which involves NADPH oxidase: an enzyme that also produces ROS and triggers innate immunity [14].

Phagocytic cells expend energy for endocytosis. The altered energy metabolism of neutrophils during phagocytosis has been previously reported [15], and the efficiency of phagocytosis decreases at low temperatures [16], as previously demonstrated in neutrophils [8]. Adenosine triphosphate (ATP) is the primary energy source used by cells and is generated through glycolysis and oxidative metabolism in the mitochondria; ATP synthase (complex V) in the mitochondrial membrane electron transport chain produces ATP. Furthermore, previous research links oxygen consumption and mitochondrial respiration to phagocytosis and cytokine production [17].

Nuclear factor-κB (NF-κB) is a transcription factor that plays an important role in immunity. Classical NF-κB in the cell is a heterodimer consisting of one p50 subunit and one p65 subunit that is rapidly activated in response to a wide variety of stimuli, including pathogens, stress signals, and pro-inflammatory cytokines [18,19]. The inhibitor of NF-κB (IκB) binds to NF-κB in the cytoplasm and keeps it in an inactive state. Phosphorylation of p65 and IκBs by IκB kinases (IKKs) activates NF-κB. Phosphorylated IκBα is ubiquitinated and degraded by proteasomes. After IκBα is released from the NF-κB complex, NF-κB translocates to the cell nucleus and promotes the transcription of specific inflammatory cytokines such as IL-6 and TNF-α. Additionally, endocytosis activates the inflammasome [20]. The production of IL-1β, another inflammatory cytokine, needs two steps: the first is gene activation, followed by final activation through caspase-1 after inflammasome formation. 

Endocytosis, energy consumption, and cytokine release are intricately intertwined in some types of immune cells. However, these events have not yet been investigated in neutrophils. In this study, we characterized the relationship between endocytosis of biological PM and energy consumption in neutrophils.

## 2. Results

### 2.1. Dynamin Inhibitors Suppress Endocytosis-Induced ATP Consumption in Neutrophils

**Figure 1:**
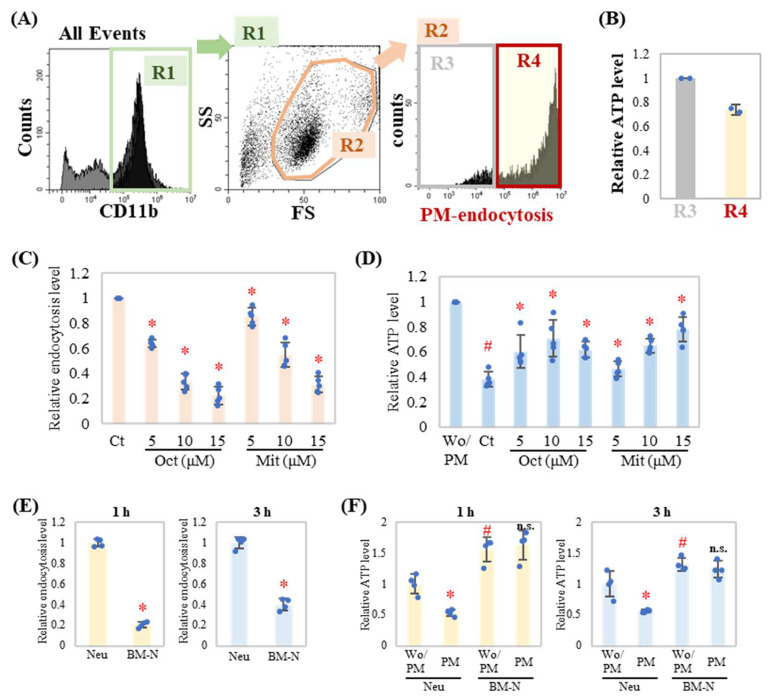
Dynamin inhibitor relieved endocytosis-induced ATP consumption in neutrophils. (**A**) Gating strategy: Neutrophils were cultured with PM for 3 h, and then stained with an anti-CD11b antibody and analyzed by flow cytometry. Total events were separated into two regions. The cells in the right region were CD11b positive cells (R1). The R1 population was plotted by side scatter and forward scatter. The R2 population represents CD11b-expressing neutrophils. R2 was further separated into two regions. The cells in the R4 region represent PM-endocytosed neutrophils. (**B**) The neutrophils in the R3 and R4 populations were sorted using a cell sorter. Intracellular ATP levels of cells from R3 and R4 were measured with a luminometer, and results were normalized to cell number (*n* = 2). The results are expressed as the mean ± SD relative to the ATP level of the R3 region. (**C**,**D**) Neutrophils were treated with dynamin inhibitors OctMAB (Oct) or MitMAB (Mit) with indicated concentration for 1 h then cultured with PM (20 μg/mL) for 3 h (*n* = 4). (**C**) Relative endocytosis level (R4 region) was evaluated. For relative endocytosis level, PM treatment with dimethyl sulfoxide (DMSO, solvent for inhibitors) group (Ct) was set as the reference group (1.0). (**D**) Relative ATP level was evaluated. (**E**,**F**) Ly6G positive cells (BM-N) were separated from mouse bone marrow cells by a magnet separation system. Neutrophils and BM-N were cultured with PM for 1 h or 3 h. (**E**) Relative endocytosis level (R4 region) was evaluated. For relative endocytosis level, Neutrophils treated with the PM group were set as the reference group (1.0). (**F**) Relative ATP level was evaluated. Representative analyses from two independent experiments are shown. Results were shown as dots, mean relative level (indicated by columns for each group), and SD of each treatment. For relative ATP level, no treatment (Wo/PM) was set as the reference group (1.0). Neu, neutrophils. BM-N, Ly6G positive cell from mouse bone marrow. * *p* < 0.05 vs. Ct. # *p* < 0.05 vs. Wo/PM (Neu). n.s., not significant vs. Wo/PM (BM-N).

To evaluate cellular energy consumption during PM endocytosis in neutrophils, cellular ATP levels were measured using the Cell Titer Glo Luminescent Cell Viability Assay. Flow cytometry analysis was performed to reveal PM endocytosis (Figure 1A, R4). Endocytosis with PM reduced cellular ATP levels (Figure 1B). We have previously reported that dynamin inhibitors suppress PM endocytosis in neutrophils [8]. In concert with the inhibition of endocytosis, the addition of dynamin inhibitors OctMAB and MitMAB relieved ATP consumption in neutrophils in a dose-dependent manner (Figure 1C,D). These results suggest that endocytosis of PM by neutrophils is associated with cellular ATP levels. Different neutrophil sources were compared using the same experiments that were performed using bone marrow-derived neutrophils [BM-N, lymphocyte antigen 6 complex locus G6D (Ly6G) positive cell]. Results showed that the PM endocytosis of BM-N was lower than that of neutrophils (Figure 1E). The cellular ATP level of BM-N was higher than that of neutrophils (Figure 1F).

### 2.2. Inhibition of ATP Synthase Disrupts Endocytosis in Neutrophils

**Figure 2:**
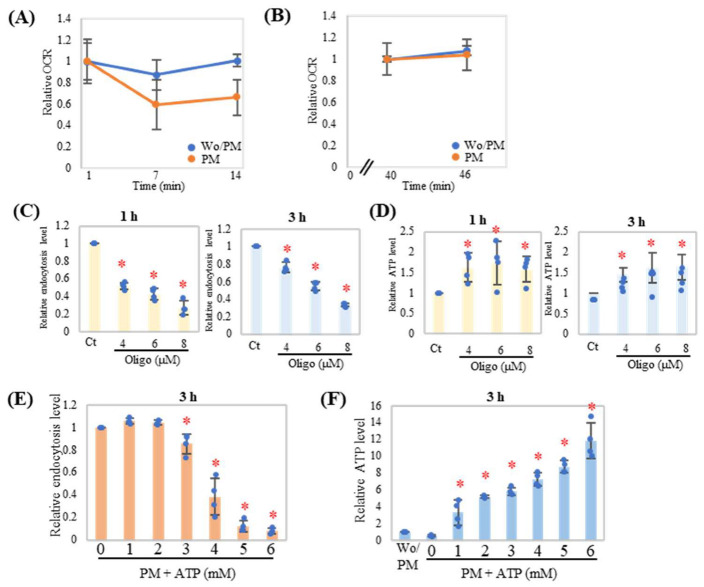
Inhibition of ATP synthase interrupts endocytosis in neutrophils. (**A**,**B**) Neutrophils were treated with or without PM (20 μg/mL) for 1 h. Then, oxygen consumption rate (OCR) was measured using Seahorse XF Analyzers. Relative OCRs were calculated from 3 wells. The non-treated group (Wo/PM) was set as the reference group (1.0). (**C**–**F**) Neutrophils were treated with indicated concentration of oligomycin (**C**,**D**) or ATP (**E**,**F**) for 1 h then cultured with PM (20 μg/mL) for 1 h and/or 3 h (*n* = 4). Representative analyses from two independent experiments are shown. Relative endocytosis levels (**C**,**E**) and relative ATP levels (**D**,**F**) were evaluated. The results were shown as dots, mean relative level (indicated by columns for each group), and SD of each treatment. For relative level, PM treatment with DMSO (Ct) was set as the reference group (1.0). For (**F**), the non-treated group (Wo/PM) was set as the reference group (1.0). * *p* < 0.05 vs. Ct.

As shown in Figure 1, endocytosis is related to cellular ATP levels. Therefore, we evaluated oxygen consumption by neutrophils and measured the oxygen consumption rate (OCR), which is related to ATP production, using an extracellular flux analyzer. The OCR of PM-treated neutrophils was lower than that of the control group (Figure 2A), suggesting that ATP consumption and low oxygen conditions occurred during endocytosis. After treatment with oligomycin, the OCR was comparable between the control and Bio-PM-treated groups (Figure 2B), suggesting that endocytosis utilizes ATP to the maximum capacity. Accordingly, inhibition of ATP synthase by oligomycin reduced PM endocytosis in a dose-dependent manner (Figure 2C). Furthermore, oligomycin inhibited cellular ATP consumption during PM endocytosis (Figure 2D). To further evaluate the effects of ATP on endocytosis, neutrophils were treated with different doses of exogenous ATP. In the presence of exogenous ATP, neutrophils exhibit different behaviors during endocytosis based on the ATP treatment concentration. When the ATP concentration was lower than 2 mM, endocytosis was not affected; however, when the ATP concentration was higher than 4 mM, endocytosis dramatically decreased (Figure 2E,F) without cytotoxicity (Supplementary Figure S1).

### 2.3. Inhibition of NADPH Oxidase Suppresses Late-Phase Neutrophil Endocytosis

**Figure 3:**
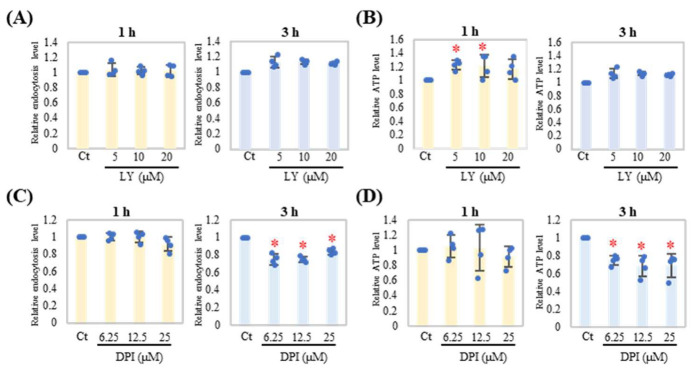
Inhibition of NADPH oxidase resulted in suppression of late-phase neutrophil endocytosis. Neutrophils were treated with the indicated concentration of LY294002 (LY, (**A**,**B**)) or diphenyleneiodonium chloride (DPI, (**C**,**D**)) for 1 h then cultured with PM (20 μg/mL) for 1 h or 3 h (*n* = 4). Endocytosis (**A**,**C**) and ATP level (**B**,**D**) were evaluated in the same manner as Figure 1. Representative analyses from two independent experiments are shown. Results were shown as dots, mean relative level (indicated by columns for each group), and SD of each treatment. For relative levels, PM treatment with DMSO (Ct) was set as the reference group (1.0). * *p* < 0.05 vs. Ct.

PI3K and NADPH oxidases play important roles in phagosome formation. Here, the type 1 PI3K inhibitor LY294002 (LY) did not inhibit endocytosis, whereas cellular ATP levels slightly changed during the early phase of endocytosis (1 h) (Figure 3A,B). In contrast, diphenyleneiodonium chloride (DPI), an inhibitor of NADPH oxidase, inhibited endocytosis and decreased cellular ATP levels in the late phase (3 h) (Figure 3C,D).

### 2.4. Endocytosis Inhibition by IKK Inhibitor-Induced ATP Accumulation in Neutrophils

**Figure 4:**
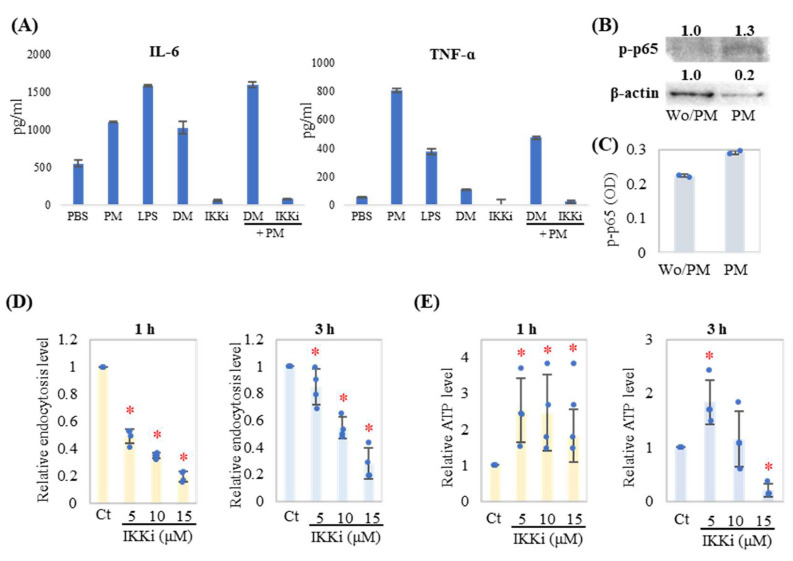
IKK inhibition suppresses endocytosis and induces ATP accumulation in neutrophils. (**A**) Neutrophils were incubated with PM (20 μg/mL) or LPS (1 μg/mL) for 3 h following preincubation with/without the IKK inhibitor VII (IKKi, 10 μM) for 1 h. Cytokines (IL-6 and TNF-α) in cell culture supernatant were measured by enzyme-linked immunosorbent assays (ELISA). DM = DMSO (control for IKKi). (**B**) Phosphorylated (p)-p65 (p-p65 at Serine 536) was detected by western blotting. β-actin served as the loading control. Relative densities of bands are shown above each respective band. Densities from the non-PM treatment (Wo/PM) band were used as the reference (1.0) to calculate relative band intensities. (**C**) The amount of p-p65 was quantified by ELISA. An equal protein amount (5 μg) was loaded in each well. (**D**,**E**) Neutrophils were treated with the indicated concentration of IKKi for 1 h and then cultured with PM (20 μg/mL) for 1 h or 3 h (*n* = 4). Representative analyses from two independent experiments are shown. Relative endocytosis level (**D**) and relative ATP level (**E**) were evaluated and shown as dots with the mean relative level (indicated by columns for each group) and SD of each treatment. For relative endocytosis level and relative ATP level, PM treatment with DMSO (Ct) was set as the reference group (1.0). * *p* < 0.05 vs. Ct.

We have previously reported that dynamin inhibitors can inhibit PM endocytosis-induced inflammatory cytokine production in neutrophils. NF-κB is a master regulator of inflammatory cytokine production. To assess NF-κB involvement in neutrophil endocytosis, an IKK inhibitor was applied to this system. Endocytosis of neutrophils induces IL-6 and TNF-α production, which was inhibited by IKK inhibitor treatment (Figure 4A). Western blot showed that one NF-κB component, p65, was activated (phosphorylated) during endocytosis (Figure 4B,C). As expected, the IKK inhibitor treatment suppressed endocytosis in a dose-dependent manner (Figure 4D) and induced cellular ATP accumulation in neutrophils (Figure 4E), while inhibitors themselves do not inhibit cellular ATP levels (Supplementary Figure S2).

### 2.5. Proteasome Inhibitors Do Not Disrupt Endocytosis in Neutrophils

**Figure 5:**
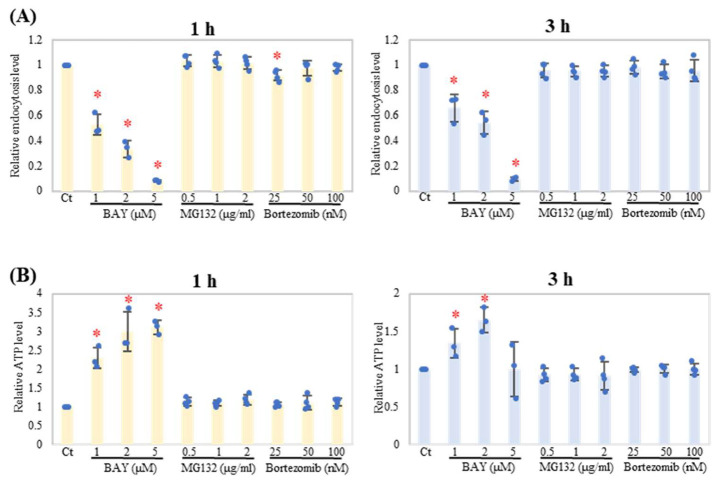
Proteasome inhibitors do not interrupt endocytosis in neutrophils. Neutrophils were treated with the indicated concentration of BAY 11-7085 (BAY), MG132, or Bortezomib for 1 h before treatment with PM (20 μg/mL) for 1 h or 3 h (*n* = 4). Relative endocytosis level (**A**) and relative ATP level (**B**) were evaluated and shown as dots with the mean relative level (indicated by columns for each group) and SD of each treatment. Representative analyses from more than 3 independent experiments are shown. For relative endocytosis level and relative ATP level, PM treatment with DMSO (Ct) was set as the reference (1.0). * *p* < 0.05 vs. Ct.

To further interrogate the role of NF-κB signaling in neutrophil endocytosis, the effects of other NF-κB-related inhibitors were tested. The IKK inhibitor BAY 11-7085 (BAY) suppressed endocytosis in a dose-dependent manner at both 1 and 3 h of treatment in neutrophils (Figure 5A). As observed after IKKi treatment, cellular ATP levels in neutrophils were increased by BAY treatment (Figure 5B). On the other hand, proteasome inhibitors (MG132 and Bortezomib), which also serve as NF-κB inhibitors, affected neither endocytosis levels nor cellular ATP levels (Figure 5).

### 2.6. Inflammasome Activation Is Not Associated with ATP Consumption Induced by Endocytosis in Neutrophils

**Figure 6:**
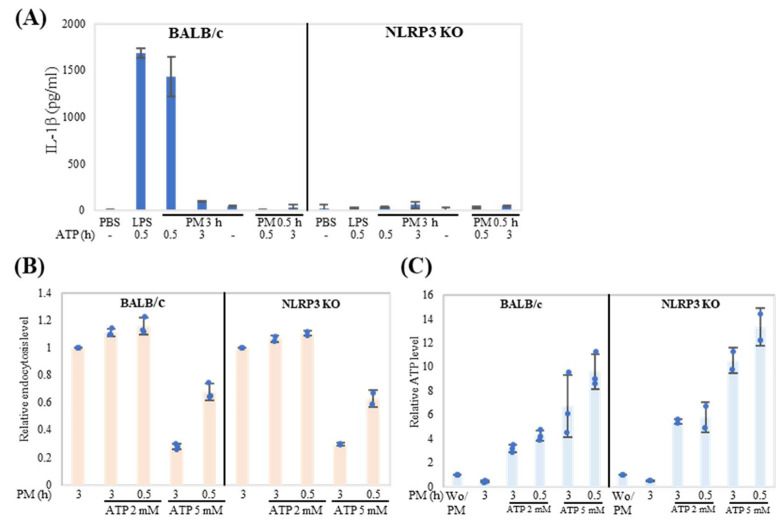
Inflammasome activation is not associated with ATP consumption induced by endocytosis in neutrophils. (**A**) Neutrophils from BALB/c and NLRP3 KO mice were incubated with PM (20 μg/mL) or LPS (1 μg/mL) for 3 h in the presence or absence of ATP (2 mM) for 3 h. The last 0.5 h treatment before harvest represents 0.5 h. IL-1β in cell culture supernatant was measured by ELISA. (**B**,**C**) Neutrophils from BALB/c and NLRP3 KO mice were incubated with PM (20 μg/mL) for 3 h in the presence or absence of ATP (2 mM or 5 mM) for 3 h. The 0.5 h group represents the last 0.5 h treatment before harvest. Relative endocytosis level (**B**) and relative ATP level (**C**) were evaluated. The results were shown as dots with mean relative level (indicated by columns for each group) and SD of each treatment. For relative levels, PM treatment with DMSO was set as the reference group (1.0). For (**C**), the non-treated group (Wo/PM) was set as the reference (1.0). Representative analyses from two independent experiments are shown.

Finally, we investigated the role of inflammasome activation in endocytosis. IL-1β production is a good marker for evaluating inflammasome activation. LPS and ATP treatment induced IL-1β production in neutrophils from BALB/c mice but not from NLRP3 knockout mice (Figure 6A). In addition, endocytosis without ATP treatment did not induce IL-1β production, suggesting that endocytosis induced the IL-1β gene but did not activate inflammasome. Next, endocytosis and cellular ATP levels were evaluated. Endocytosis was enhanced by 2 mM ATP treatment, but inhibited by 5 mM ATP treatment. There were no dramatic differences in endocytosis and cellular ATP levels between BALB/c and NLRP3 knockout (KO) mice, suggesting that inflammasome activation was not involved in ATP consumption induced by endocytosis in neutrophils (Figure 6B,C).

## 3. Discussion

The World Health Organization warned that PM-related health hazards are a serious global problem [21]. In general, PM inhalation causes lung inflammation and contributes to several chronic diseases in humans. For instance, we previously reported that PM2.5 and PM2.5-enriched air pollutants cause neutrophilic alveolitis and bronchitis [1,22]. Neutrophils are important for the initiation of inflammation through the production and secretion of various cytokines. However, the intracellular driving forces for PM endocytosis and their relationship with cytokine production and inflammasome activation in neutrophils are not fully understood. In the current study, a decrease in transient and significant intracellular ATP levels was observed during neutrophil endocytosis of *Staphylococcus aureus* particulate matter (PM). We demonstrated for the first time that the uptake of PM by neutrophils resulted in more efficient endocytosis than that of previously used silica particles. This mimics the process at the time of initiation when neutrophils capture actual pathogens. Again, as shown in a previous study, dynamin inhibitors effectively reduced endocytosis at 1 h after PM treatment. Interestingly, the intracellular levels of ATP tended to recover (increase) from 1 to 3 h. These findings support the use of ATP during endocytosis. More interestingly, inflammation-induced neutrophils have lower intracellular ATP levels than bone marrow-derived neutrophils. This may be because they were forced to consume ATP before being recruited to the site of inflammation.

Generally, inhibition of ATP synthase by oligomycin reduces the intracellular OCR [23]. We also demonstrated that OCR decreased after endocytosis in neutrophils. This may indicate that the electron transport chain temporarily reduces mitochondrial function owing to the rapid consumption of ATP by endocytosis. Oligomycin treatment erased the difference in the OCR observed between PM endocytosis cells and the inhibitor alone, suggesting that PM endocytosis causes a dramatic decrease in oxygen consumption. The intracellular ATP level was decreased by PM in oligomycin-treated cells and seemed to recover. Interestingly, exogenous ATP dramatically reduced cellular endocytosis at higher concentrations. We observed that this was not ATP-induced cytotoxic cell death. In addition, no IL-1β production was observed at high concentrations (5 mM) of ATP (Supplementary Figure S3). Regulation of ATP concentration similar to this feedback inhibition is very interesting, but further detailed analysis is needed.

We demonstrated that dynamin inhibition increased ATP levels. This was due to the lack of endocytosis initiation without ATP consumption. Many neutrophil functions, including phagocytosis, are mediated by phosphatidylinositol (3,4,5)-trisphosphate (PIP3) [14]. The dynamin PH domain is important for endocytosis, and phosphatidylinositol lipids bind to the PH domain of biological membranes [24]. PIP3 is generated by the phosphorylation of PIP2 by phosphatidylinositol-3-kinase (PI3K). This process can be inhibited by PI3K inhibitors, such as LY294002, which mainly inhibits class I PI3K [25]. During endocytosis, phagosome formation requires PIP3 and the NADPH oxidase complex [14]. However, LY did not inhibit endocytosis after 1 h of PM treatment, suggesting that PI3K does not affect phagosome formation in PM endocytosis at an early stage; the first step of phagosome formation occurs very quickly. NADPH oxidase inhibitors showed an effect only after a short period (3 h). Because the endosome closes very quickly, it may not have been affected by inhibitors before PM was taken up and the endosomes closed. It is possible that the inhibition of ROS production mediated by NADPH oxidase affected endocytosis as well. Indeed, in our previous study, we observed that ROS quenching by N-acetyl cysteine enhanced endocytosis, which is related to the observations from our current study [8].

Upon endocytosis, neutrophils rapidly produce inflammatory cytokines [8,26]. NF-κB signaling mainly acts on the production of IL-6 and TNF-α through the upregulation of the transcriptional activity of these genes [27]. Accordingly, treatment of neutrophils with IKK inhibitor VII, which inhibits NF-κB signaling, before stimulation with PM completely abrogated the production of these cytokines. In addition, when neutrophils undergo endocytosis, intracellular phosphorylation of p65—a component of NF-κB—is observed. Consistent with these results, IKK inhibitor VII inhibited endocytosis in a dose-dependent manner and tended to restore ATP levels. However, this condition did not last long; at 3 h, it decreased at high inhibitor concentrations. Collectively, these results suggest that endocytosis and ATP consumption simultaneously occurred. Interestingly, IKK inhibitor VII has been shown to competitively inhibit IKK when ATP is used for signaling [28,29]. From this perspective, the inhibitory effect is convincing. In contrast, our results showed that the inhibition of NF-κB signaling did not uniformly inhibit endocytosis. BAY 11-7085, which also inhibits IKK, showed the same tendency as IKK inhibitor VII. BAY 11-7085 has been shown to exhibit the blocking of ATPase activity [30]. At the initialing phase of endocytosis, the neutrophil membrane needs to change shape to endocytose PM. It was reported that this process needs ATPase activity [31]. As mentioned before, BAY 11-7085 could block ATPase activity. It affected cell membrane shape change at the early stage of endocytosis, which could inhibit neutrophil endocytosis. On the other hand, neutrophil endocytosis of PM induced TNF-α production and extracellular TNF-α can enhance neutrophil endocytosis of PM as our previous report [8]. It was reported that BAY 11-7085 could inhibit TNF-α production [32], which will affect endocytosis levels. However, subsequent inhibition of proteasome activity (MG132 and Bortezomib) did not inhibit endocytosis. PI3K has also been reported to inhibit NF-κB signaling [33]. However, LY had no effect on endocytosis, suggesting that signaling upstream of IKK was not involved in endocytosis. Events after the activation of NF-κB itself and different upstream IKK signaling pathways may not be related to endocytosis. Considering this information, targeting ATP competitors as drugs to suppress severe inflammation, such as cytokine storms, will be a valuable future perspective.

**Figure 7:**
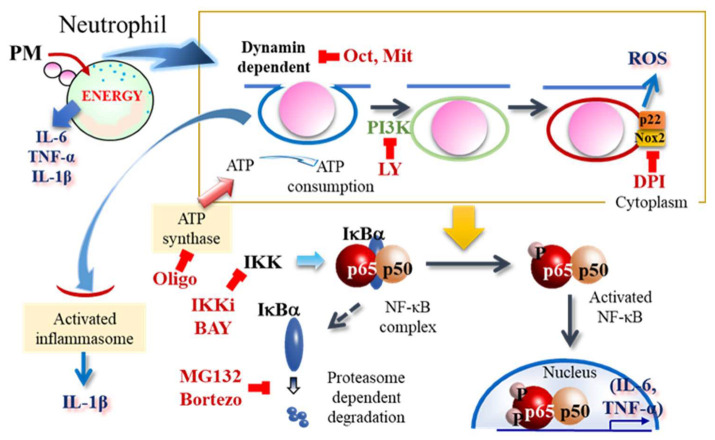
Schematic of neutrophil PM endocytosis using high doses of energy. Dynamin is important for endocytosis. PI3K can help close endosomes. The NADPH oxidase complex (NOX2, p22) associates with endosomes and generates ROS. ATP is the most important energy source to complete all steps of endocytosis; most cellular ATP is generated by ATP synthase in the mitochondria. Endocytosis induces activation of NF-κB through phosphorylation of p65 by IKK. For feedback regulation of NF-κB, the proteasome system is important; IκBα released from the NF-κB complex is degraded by the proteasome. ATP is important for inflammasome activation, which is related to IL-1β production. Overall, endocytosis of PM produces inflammatory cytokines such as IL-6, TNF-α, and IL-1β using ATP pools as the primary energy source. Dynamin inhibitors; Oct: OctMAB, Mit: MitMAB, class I PI3K inhibitor; LY: LY2904002, NADPH oxidase inhibitor; DPI: dibenziodolium chloride, ATP synthase inhibitor; Oligo: oligomycin, IKK inhibitors; IKKi: IKK inhibitor VII, BAY: BAY 11-7085, proteasome inhibitors; MG132, Bortezo: Bortezomib.

IL-1β production requires two signals. First, gene expression of IL-1β is induced and then the inflammasome is activated [34]. ATP signaling from the P2X7 receptor is often used to activate this inflammasome [35]. Dong et al. reported that inflammasome activation and phagocytosis are closely associated with bone marrow-derived macrophages [36]. Finally, we investigated the relationship between the inflammasomes and neutrophil phagocytosis. In this study, we found that phagocytosis of PM induces the expression of IL-1β itself but does not activate the inflammasome. Interestingly, IL-1β secretion was not induced when the second signal, ATP, was treated for a longer time (3 h). This result is consistent with that reported by Son [20]; however, the detailed mechanism remains unknown. In contrast, endocytosis occurred in NLRP3 KO mice, which lacked inflammasome activation. In both wild-type mice and NLRP3 KO mice, low concentrations of ATP (2 mM or less) tended to increase endocytosis or have no effect, whereas high concentrations (4 mM or more) tended to inhibit endocytosis. In contrast to the findings of Dong et al., inflammasome activation was not associated with endocytosis in neutrophils. Of note, neutrophils and macrophages may have different mechanisms of phagocytosis, even though they are the same phagocytic cells. Son et al. proposed that, unlike macrophages, inflammasomes persist in neutrophils. The phenomenon demonstrated here is also interesting, and the involvement of NLRP3 may be observed by altering the timing of endocytosis and inflammasome occurrence. In the actual living body, after catching the pathogen, neutrophils cause self-destruction, known as NETosis. After the exhaustion of ATP during endocytosis, NETosis may be induced in neutrophils. These events are depicted in Figure 7.

## 4. Materials and Methods

### 4.1. Mice and Preparation of Neutrophils

BALB/c mice (7–11 weeks) were purchased from Japan SLC Inc. (Hamamatsu, Japan). NLRP3 knockout (KO) mice (BALB/c background) were generated using the Crisper Cas9 system. Neutrophils were obtained from mice intraperitoneally injected (i.p.) with 2 mL of 4% thioglycolate broth (Laboratorios Conda S.A., Madrid, Spain), as previously reported [8]. Briefly, after 4 h i.p., mice were anesthetized using Sevoflurane (Fujifilm Wako Pure Chemical Corporation, Osaka, Japan). After 2 min, the mice were sacrificed and cells from the peritoneal exudate were harvested and suspended. CD11b-positive cells (R1 in Figure 1) and high-side and forward-scatter regions (R2 in Figure 1) were identified as neutrophils.

### 4.2. Cell Culture Conditions and Cell Separation

Neutrophils were cultured in Roswell Park Memorial Institute (RPMI) 1640 medium (Nissui, Tokyo, Japan) supplemented with 10% fetal bovine serum (HyClone, Marlborough, MA, USA), L-glutamine (2 mM, Fujifilm Wako Pure Chemical Corporation), and penicillin–streptomycin solution (Gibco, New York, NY, USA) in a humidified 5% CO_2_ incubator at 37 °C. Ly6G positive cells were isolated from mouse bone marrow cells using an IMag bead separation system (BD Biosciences, San Jose, CA, USA).

### 4.3. Reagents and Antibodies

Biological particulate matter (PM), which is derived from pHrodo™ Red Staphylococcus aureus Bioparticles™ Conjugate for phagocytosis (A10010), was purchased from Invitrogen (Woltham, MA, USA). Oligomycin (ab141829), the PH domain inhibitor MiTMAB (ab120466) and OctMAB (ab120467) were purchased from Abcam (Cambridge, UK). DPI (81050) was purchased from Cayman Chemical Co. (Ann Arbor, Michigan, USA). PerCP Cy5.5-conjugated anti-human CD11b (65–0112-U100) was purchased from Tombo Biosciences (San Diego, CA, USA). Anti-mouse Ly6G particles (558111) were purchased from BD Biosciences (San Jose, CA, USA). Adenosine triphosphate ATP (017-21511) was purchased from Wako (Osaka, Japan). The IKK inhibitor VII (401486) was purchased from Calbiochem (Darmstadt, Germany). BAY 11-7085 (BML-EI279) was purchased from Enzo Life Sciences (Farmingdale, NY, USA). LY294002 (#9901S) was purchased from Cell Signaling Technology (Danvers, MA, USA). These inhibitors were dissolved in dimethyl sulfoxide (DMSO) and diluted in PBS before treatment. Bortezomib (7282) was purchased from Tocris (Minneapolis, MN, USA). MG-132 (474790) was purchased from Calbiochem. Bortezomib and MG-132 were dissolved in ethanol and diluted in PBS before treatment.

### 4.4. Flow Cytometry

Cells (5 × 10^4^) were treated with PM for 3 h with or without a prior 1 h treatment with inhibitors at 37 °C in a 5% CO_2_ incubator. Then, the cells were harvested and the PerCP Cy5.5-conjugated anti-CD11b antibody was added and incubated for 30 min at 4 °C. Cells were washed. The PM was labeled with pHrodo™ dye and was endocytosed and encapsulated in vesicles. As the vesicles were processed, the pH decreased, and the pHrodo™-labeled particles showed bright fluorescence. Then, cells were analyzed using a CytoFLEX flow cytometer (Beckman Coulter, Brea, CA, USA).

### 4.5. Cell Sorting

The cells were treated with PM for 3 h and then harvested. Subsequently, the PerCP Cy5.5-conjugated anti-CD11b antibody was added and incubated for 30 min at 4 °C. The cells were washed and sorted using a BD FACS Melody cell sorter (BD Biosciences, San Jose, CA, USA) with the indicated gating strategy.

### 4.6. Measurement of Cellular Adenosine Triphosphate (ATP) Level

ATP levels were measured using the Cell Titer Glo luminescent cell viability assay (Promega, Madison, WI, USA) as previously reported [37]. After stimulation, the cells were collected and centrifuged, and the supernatant was discarded and resuspended in RPMI1640 medium. The supernatant was then transferred to a 96-well black plate to measure the ATP level. The substrate, including the cell lysis buffer, was added to each well (10 μL/well). Luminescence was measured using a luminometer (Fluoroskan™ FL Microplate Fluorometer and Luminometer, Thermo Scientific, Waltham, MA, USA).

### 4.7. Extracellular Flux Analysis

An XF96 Extracellular Flux analyzer (Seahorse Bioscience, North Billerica, MA, USA) was used to quantify the OCR as previously reported [23]. Neutrophils were treated with or without PM (20 μg/mL) for 1 h, then cells were plated on XF96 cell culture microplates (4 × 10^4^ cells/well) coated with Cell-Tak (BD Biosciences, San Diego, CA, USA). OCR was measured in Seahorse XF medium under basal conditions (Agilent Technologies, Santa Clara, CA, USA). Oligomycin (2 µM) was added at the indicated time points.

### 4.8. Enzyme-Linked Immunosorbent Assay (ELISA)

Enzyme-linked immunosorbent assay kits for mouse IL-6 and TNF-α were purchased from BioLegend (San Diego, CA, USA). Neutrophils were incubated with PM (20 μg/mL) or LPS (1 μg/mL) for 3 h following preincubation with/without IKK inhibitor VII (10 μM) for 1 h. Culture supernatants were collected and assayed for cytokine levels according to the manufacturer’s protocol and a previous report [38]. ELISA kits for p-p65 (Ser536 and #7834) were purchased from Cell Signaling Technology. The amount of p-p65 was quantified by ELISA, and the optical density (OD) was measured following the manufacturer’s protocol. Equal amounts of protein (5 μg) were added to each well. Culture lysates were collected and assayed for p-p65.

### 4.9. Western Blotting

Cells were lysed with radioimmunoprecipitation assay buffer to prepare whole-cell extracts [39]. Equivalent amounts of protein (5 μg) were subjected to electrophoresis. The signal intensity was quantified using ImageJ software (ImageJ bundled with 64-bit Java 1.8.0_112; National Institute of Health, Bethesda, MD, USA). The expression levels of the target proteins were normalized to that of β-actin.

### 4.10. Statistics

Results are expressed as means ± standard deviations. Each dot indicates the number of experimental mice, and each column represents the mean level of each group. Statistical analyses were performed using Fisher’s least significant difference (LSD) test using a one-way analysis of variance. Statistical significance was set at *p* < 0.05.

## Data Availability

The data presented in this study are available upon request from the corresponding author.

## Supplementary Materials

The supporting information can be downloaded at: https://www.mdpi.com/article/10.3390/ijms24109039/s1.
