# Supplementary material for: ATP Consumption Is Coupled with Endocytosis in Exudated Neutrophils
Source: Int J Mol Sci. 2023 May 20;24(10):9039. doi: 10.3390/ijms24109039 (PMC10219472; doi:10.3390/ijms24109039)
Supplement: Supplementary file 1 [file ijms-24-09039-s001.zip › ijms-2263263-supplementary.pdf]

Supplement Figure S1

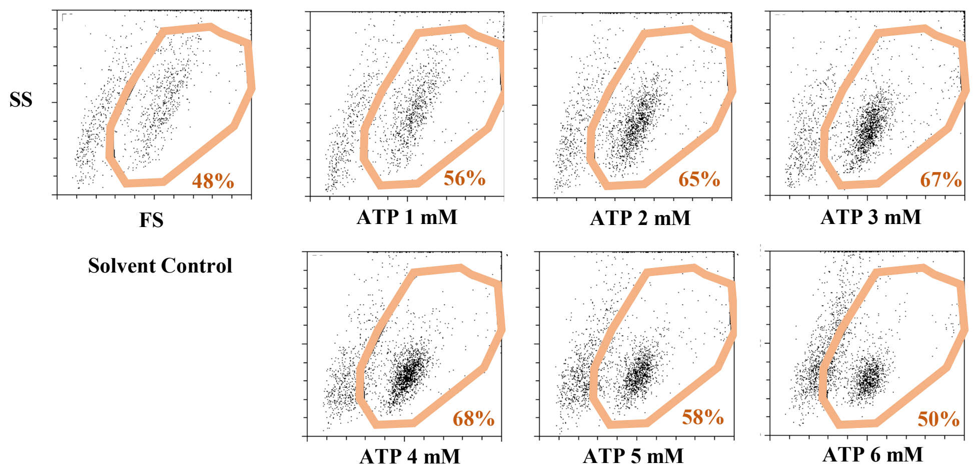

Neutrophils were treated with indicated concentration of ATP for 1 h then cultured with PM (20 μg/ml) for 3 h. Then stained with an anti-CD11b antibody and analyzed by flow cytometry. CD11b positive cells were shown and plotted by side scatter (SS) and forward scatter (FS). Percentage showed CD11b positive intact neutrophils.

Supplement Figure S2

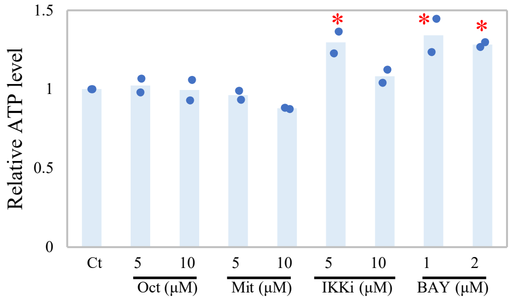

Neutrophils were treated with indicated concentration of inhibitors for 2 h. Relative ATP level was evaluated (n = 2). Results were shown as dots, mean relative level (indicated by columns for each group). Solvent control treatment group was set as the reference group (1.0). *$p < 0.05$ vs. Ct.

Supplement Figure S3

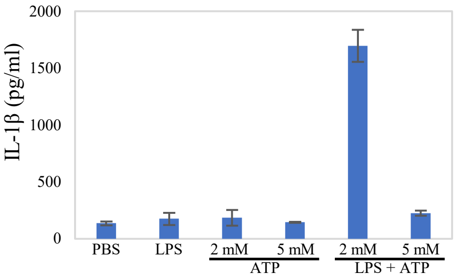

IL-1β production was low when treated with high concentration of ATP. Neutrophils were incubated with LPS (1 μg/ml) for 3 h in the presence or absent of ATP (2 mM or 5 mM). ATP was added last 0.5 h before harvest. IL-1β in cell culture supernatant were measured by enzyme-linked immunosorbent assays (ELISA).
